# Choroidal vascular changes in age-related macular degeneration: A protocol for systematic review and meta-analysis

**DOI:** 10.1097/MD.0000000000023200

**Published:** 2020-11-13

**Authors:** Xiaoqin Wang, Liuzhi Zeng, Ming Chen, Longqian Liu

**Affiliations:** a Department of Ophthalmology, West China Hospital, Sichuan University; b Department of Ophthalmology, Chendu First Peoples’ Hospital, Chengdu, Sichuan Province, China.

**Keywords:** age-related macular degeneration, choroidal thickness, choroidal vascularity index, optical coherence tomography

## Abstract

**Background:**

As an increasing age-related eye disease, age-related macular degeneration (AMD) is becoming a common cause of irreversible visual loss in elder population. The mechanism of AMD remains uncertain and covers a complex risk factors. Choroidal vascularity index (CVI) is a sensitive parameter obtained by enhanced depth imaging of optical coherence tomography which allows the choroid in more detail and accurate assessment in the pathogenesis of AMD. The objective of this current study is to evaluate choroidal structural alternations measured by CVI in AMD.

**Methods:**

We will review 4 English databases (PubMed, Embase, Cochrane Library, and Web of Science) from their inception until present to select eligible articles. English-language and case–control studies will be accepted. The data extraction content and quantitative analysis will be performed systematically by 2 independent authors. The primary outcome is the alternation of CVI in AMD. The secondary outcomes consist of choroidal thickness (CT), luminal area (LA), stromal area (SA), and total choroidal area (TCA). Subgroup analysis, sensitivity analysis, and publication bias will be performed to check the robustness of the pooled outcome data.

**Results:**

Changes of quantitative parameters such as CVI will be obtained in patients with AMD.

**Conclusion:**

This study will elucidate alternations of choroidal vascular and stromal component in AMD and provide robust evidence on the pathophysiology of AMD.

**Registration number:**

INPLASY.

## 1 Introduction

As an increasing age-related eye disease, age-related macular degeneration (AMD) is becoming a leading cause of irreversible visual impairment and blindness in elder population.^[1,2]^ AMD is clinically divided into 2 principle stages: early AMD (characterized by medium-sized drusen and abnormalities of retinal pigment epithelial) and late AMD (characterized by neovascularization and geographic atrophy).^[3]^ Polypoidal choroidal vasculopathy (PCV), generally seen in Asian, is a subset of neovascular AMD because they share many common genetic factors and clinical features.^[4]^ But this viewpoint is still a subject of controversy.^[5]^ The mechanism of AMD remains uncertain and covers a complex risk factors.^[6]^ It is an urgent priority to understand the pathophysiology of AMD. While the main sites of pathogenesis in AMD are focused on the lesion of Bruch's membrane and retinal pigment epithelium, choroidal structure disorders also play a vital role.^[7–9]^ Numerous studies indicate that choroid thinning occurs in AMD and choroidal thickness (CT) does not connected with sensitivity of AMD.^[10–12]^ However, the overall analysis of choroidal structure in AMD is inconclusive, whether the decrease of CT is owing to shrinkage of choroidal vessels or choroidal stroma has no consensus.^[13]^ Histopathological studies of AMD have demonstrated that choroid plays a significant role in pathogenesis, but choroidal thickness cannot discover these subtle changes.^[14,15]^

Advances in swept-source spectral-domain optical coherence tomography (OCT) with enhanced depth imaging (EDI) model make a more detailed study of choroid and accurate assessment of the potential effect of choroid in the pathogenic mechanism of AMD.^[16,17]^ Nowadays, in order to quantify the choroidal structure alterations, an novel term choroidal vascularity index (CVI) is more sensitive due to the test of choroidal vascular.^[18]^ It is defined as the proportion of choroidal vascular luminal area (LA) to total choroidal area (TCA).^[19]^ TCA means the total choroidal area and it constitutes of LA and stromal area (SA). Previous studies have investigated that the CVI is reduced in AMD patients, with no difference between early stage and late stage of AMD.^[20–22]^ The lack of difference between 2 sub-types of AMD indicates that the former may be at risk of progression. Similarly, there is no significant difference in CVI between neovascular AMD and PCV obtained by 2 studies.^[23,24]^ They consider that decreased choroidal vascularity could result in choroidal ischemia, which may result in choroidal neovascularization (CNV). CVI is also significantly diminished in AMD with geographic atrophy.^[25]^ Otherwise, increased CVI was noted in wet AMD with activation of CNV and in PCV with choroidal vascular hyper-permeability.^[26,27]^

To date, the change of CVI in AMD remains unclear. However, as far as we know, there is no meta-analysis existed in this subject. Therefore, to address this gap, we plan to perform a comprehensive evidence synthesis to evaluate choroidal structural alternations measured by CVI in eyes with AMD and provide robust evidence on the pathophysiology of AMD.

## 2 Methods

### 2.1 Study registration

The present protocol was registered in the International Platform of Registered Systematic Review and Meta-Analysis Protocols (INPLASY) and the registration number is INPLASY2020100041 (https://inplasy.com/inplasy-2020-10-0041/, DOI: 10.37766/inplasy2020.10.0041). This current study will be designed on the basis of the Preferred Reporting Items for Systematic Review and Meta-Analysis (PRISMA) Protocols statement.^[28]^

### 2.2 Study selection criteria

#### 2.2.1 Types of studies

Case–control studies will be included in this meta-analysis if they use OCT scans to obtain the CVI in AMD and healthy controls. All articles in English were considered eligible. However, studies with insufficient data, animal experiment, abstracts, letters, editorials, reviews, and case-reports will be excluded.

#### 2.2.2 Types of subjects

The population of participants diagnosed with AMD will be accepted regardless of age, race, and gender.

#### 2.2.3 Types of interventions

We will only accept studies that use OCT to assess the choroidal vascular changes in patients with AMD on CVI. The control group will consist of healthy eyes in the absence of any eye diseases especially choroidal retinopathy.

#### 2.2.4 Types of outcomes

The primary outcome is the change of CVI between AMD and the controls. The secondary outcomes consist of CT, TCA, LA, and SA.

### 2.3 Search method and strategy

The 4 English databases of PubMed, Embase, the Cochrane library, and Web of Science will be involved in searching the eligible literature from their inception until present. The following keywords are searched simultaneously: (“optical coherence tomography” OR “choroidal vascularity index” OR “Choroid”) AND “age-related macular degeneration” OR “choroidal vascularity index.” Additionally, relevant studies from the reference lists will be also manual-searched and examined for retrieved studies.

### 2.4 Study collection and management

#### 2.4.1 Study selection

**Figure 1:**
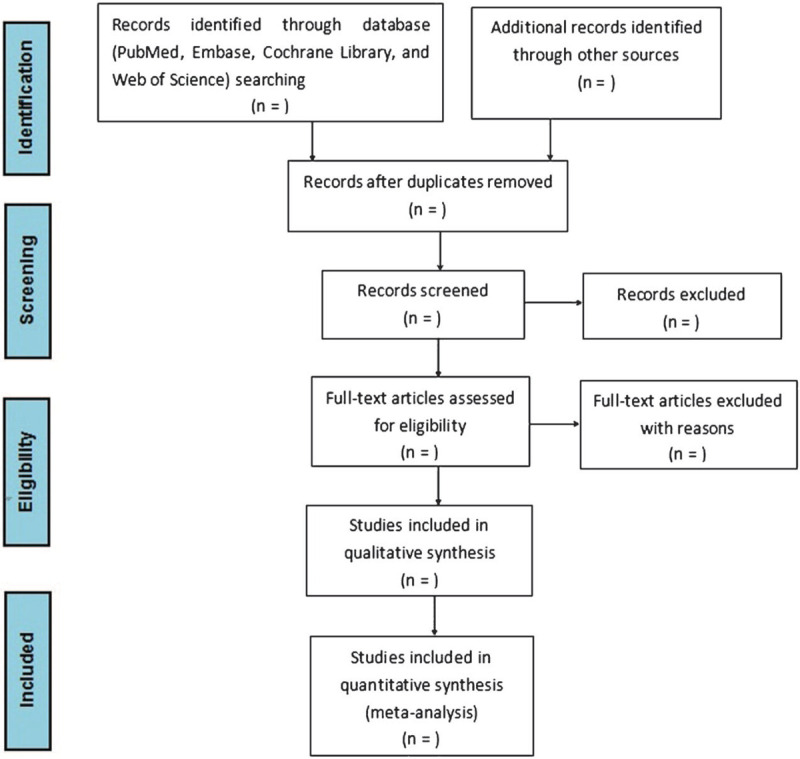
Flowchart of study search and selection.

Two investigators (XQ-W and MC) will independently review the title and abstract of all relevant articles. Then the full text will be assessed to prove whether they meet the predetermined eligibility criteria. Microsoft Excel 2016 will be used to manage the relevant data from the included studies. Discrepancies on the inclusion of data when existing will be addressed by consensus. The selection process will PRISMA guidelines and is summarized as the following diagram in Figure 1.

#### 2.4.2 Data extraction

We will meticulously filter interested results from selected studies: study setting (first author, publication year, study design, and region), characteristics of participants (age, gender, sample size, race, duration of AMD, and type of AMD), OCT instrument, and outcomes. If we cannot get the above-mentioned information, it is essential to send an email to the author for original data.

#### 2.4.3 Risk of bias assessment

Newcastle-Ottawa Scale (NOS) will be applied to evaluate the risk of bias assessment for each eligible study which includes 8 assessment items.^[29]^ The total scores ranging from 0 to 9 points apply to the evaluation and results will be presented on a table. Studies with NOS scores > 7 will be defined as high quality and consider in the final analysis.

### 2.5 Data synthesis and analysis

#### 2.5.1 Assessment of heterogeneity

Statistical analysis will be conducted using RevMan 5.3 software. For continuous variables, outcomes will be reported as the mean ± standard deviation (SD) and the mean difference (MD) with a 95% confidence interval (CI). To obtain reliable results, heterogeneity will be evaluated using the *I*^2^ statistic. If the homogeneity test shows *P* ≥ .1 and *I*^2^ ≤ 50%, which indicates a low homogeneity between the included studies, a fixed-effects model will be utilized to analyze the data. If the value of *I*^2^ is >50% or *P* value <.1, which means a high heterogeneity, then a random-effects model will be applied to pool the data. At the same time, subgroup analysis will be conducted to explore the possible causes. A *P* value <.05 is defined as statistically significant.

#### 2.5.2 Subgroup analysis

If there is apparent clinical heterogeneity and sufficient data, subgroup analysis will be conducted based on patient characteristics and OCT device brand such as duration of AMD, sub-type of AMD, and so on. However, if significant heterogeneity is still identified after subgroup analysis, data will not be recommended to pool and meta-analysis will not be performed. Instead, a narrative summary will be reported ultimately.

#### 2.5.3 Sensitivity analysis

We will carry out sensitivity analysis to assess the robustness of the pooled data for the main outcomes.

### 2.6 Publication bias

In addition, when there are more than 10 eligible articles, Egger's regression test and funnel plot will be employed to assess potential bias of publication.

### 2.7 Ethics and dissemination

No ethical approval is necessary, as the data is extracted from published studies.

## 3 Discussion

Up to the current, AMD is a multi-factorial disease with limited treatments, and its pathogenesis is not yet thoroughly elucidated.^[30]^ EDI-OCT is a vital technique for non-invasive imaging of choroid and better study the basic mechanism of chorioretinopathy disease.^[31]^ CVI is a valuable quantitative parameter in the evaluation of patients with AMD for detailed choroidal features.^[15]^ In this comprehensive systematic review and meta-analysis, we will specifically identify articles to evaluate CT, TCA, LA, SA, and CVI in patients with AMD. The results of this study will exhibit summarized data and elucidate changes in choroidal vascular and stromal component. Overall, we expect our work can help ophthalmologists with valuable insight into the pathogenesis of AMD and further and draw wide attention for both patients and researchers.

Of course, this present article has some limitations as well. Firstly, only literature published in English will be selected in this analysis. Secondly, different areas and sub-types of AMD may contribute risk of heterogeneity. Finally, the measurement of CVI and binarization do not take account of neovascular membrane in the segmented area, and further algorithm is recommended to involve in the neovascular membrane.^[23]^

## Author contributions

**Conceptualization:** Xiaoqin Wang.

**Data curation:** Xiaoqin Wang, Ming Chen.

**Formal analysis:** Liuzhi Zeng.

**Funding acquisition:** Liuzhi Zeng.

**Methodology:** Xiaoqin Wang, Ming Chen.

**Writing – original draft:** Xiaoqin Wang.

**Writing – review & editing:** LongQian Liu, LiuZhi Zeng, Ming Chen.
